# Supportive but “worried”: perceptions of naturopaths, homeopaths and Chinese medicine practitioners through a regulatory transition in Ontario, Canada

**DOI:** 10.1186/s12906-015-0846-6

**Published:** 2015-09-07

**Authors:** Nadine Ijaz, Heather Boon, Sandy Welsh, Allison Meads

**Affiliations:** Leslie Dan Faculty of Pharmacy, University of Toronto, 144 College St. (room 514), Toronto, ON M5S 3M2 Canada; Department of Sociology, University of Toronto, Toronto, Canada

**Keywords:** Traditional complementary and alternative medicine regulation health practitioner professions

## Abstract

**Background:**

In line with recent World Health Organization recommendations, many jurisdictions are taking steps to regulate practitioners of traditional, complementary and alternative medicine (TCAM). Previous studies have examined TCAM practitioners’ generally-supportive views about professional regulation; however, little research has been conducted on TCAM practitioners’ experiences and perspectives amidst an active regulatory process. In 2006 and 2007, the province of Ontario, Canada announced it would grant self-regulatory status to three TCAM practitioner groups - homeopaths, naturopaths and Chinese medicine practitioners/acupuncturists.

**Methods:**

In 2011 and 2012, part-way through each group’s regulatory process, we surveyed all practitioners from these three groups (*n* = 1047) that could be identified from public registries and professional associations. The data presented here are derived from the sub-sample of homeopaths (*n* = 234), naturopaths (*n* = 273) and Chinese medicine practitioners/acupuncturists (*n* = 181) who provided answers to an open-ended question about their opinions of the regulatory process at the end of the survey. An inductive, thematic analysis of qualitative survey responses was conducted.

**Results:**

Survey responses affirmed a pro-regulatory stance across all groups, but revealed considerable ‘worry’ amongst practitioners as to how the regulations might be implemented. Four primary ‘worry-related’ themes emerged: a) regulation’s potential administrative and financial burden on practitioners; b) scope-related concerns; c) implementation of fair registration standards; and d) whether regulation might erode the groups’ distinctive worldviews. Some occupationally-specific concerns appeared related to each group’s particular stage of professionalization. Other ‘worries’ may be related to the relative marginality of TCAM practitioner groups within biomedically-dominant national health care systems, and the possibility that inter-professional hierarchies may be emerging between particular TCAM groups. Specific concerns around overlapping practice scopes between TCAM and other professions raised questions about the implementation of non-monopolistic regulatory models such as Ontario’s.

**Conclusions:**

Overall, this study will help inform regulators and TCAM practitioner groups to navigate the unique challenge of regulating health care providers long excluded from national health care systems, who frequently work from within paradigms distinct from mainstream biomedicine.

## Background

The World Health Organization has called for member countries to increasingly regulate practitioners of traditional, complementary and alternative medicine (TCAM), with the aim of increasing safety, quality and effectiveness of TCAM therapies worldwide [1]. Many nations continue to grapple with how to regulate TCAM practitioners and practices, which have historically fallen outside of the purview of biomedicine’s globalized dominance in government-sanctioned health care systems, and which, as such, are characterized by relative sociopolitical marginality [2]. TCAM practices are, furthermore, characterized by paradigmatic features distinct from biomedicine’s underpinnings; this poses additional regulatory challenges in that many regulatory systems have been designed to accommodate biomedical-style health care [3, 4]. Regardless, several nations - including some jurisdictions within Canada [5] - continue to make significant headway in regulating TCAM practitioner groups. Lessons learned from these jurisdictions may ease the process for others.

In some jurisdictions, governments are taking pro-active steps in support of the WHO’s call for increased integration of TCAM practitioners into their national health care systems. In 2013, for instance, several southeast Asian countries co-signed the Delhi Declaration on Traditional Medicine, agreeing to collaborate towards a ‘harmonized approach… [to] regulation of traditional medicine and involvement of traditional medicine practitioners in health services’ [6]. In many cases, however, the impulse to lobby governments to regulate TCAM practitioners has arisen from within the occupational groups themselves, as exemplified in our previous studies involving naturopaths, homeopaths and Chinese medicine practitioners in the Canadian province of Ontario [4, 7–10]. As we and others [3, 11, 12] have noted, some of the principal drivers behind such groups’ professionalization projects include: the pursuit of increased occupational credibility, more extensive inclusion within third party and/or public health insurance programs, and the promotion of higher or more consistent training standards.

Despite widespread support for professionalization - and with it, regulation - within many TCAM practitioner groups, some issues continue to prove contentious within these communities. Standardization of educational requirements can - for instance - prove challenging for groups characterized by significant inter-occupational diversity in terms of both practice style and training background [13]. While TCAM practitioner training is increasingly institutionalized worldwide, traditional apprenticeship or mentorship-based training models continue to carry importance globally [14]. How much biomedical-style training should be included in TCAM practitioners’ training continues to be contentious within some groups [10, 15]; and some practitioners fear long-term co-optation into a biomedically-dominant model [3, 16]. In some cases, these issues have proven so controversial within practitioner communities that statutory regulation has been largely abandoned as a common goal [16]. Herbalists in the United States, for instance, organize within a professional ‘guild’ model which explicitly recognizes multiple practice styles and diverse training pathways, rather than pursue state regulation [17]. Regardless, many TCAM practitioner groups, as well as an increasing number of nations, remain committed to a regulatory pursuit, in line with the WHO’s call.

### The case of naturopaths, homeopaths and Chinese medicine practitioners in Ontario, Canada

In 2006 and 2007, the Ontario (Canada) government announced that it would move forward in regulating practitioners of naturopathy, homeopathy and traditional Chinese medicine/acupuncture under the Regulated Health Professions Act [18]. Two points should be noted about Ontario’s health professions regulatory structure. First, the province’s health professions are self-regulated with government oversight, in contrast with various types of state regulation and co-regulation more typical in other jurisdictions (such as Europe and Australia). Second, the provincial government does not authorize exclusive practice scopes to particular professions (as is more common in other jurisdictions) [7, 19]. In Ontario’s ‘overlapping-scopes’ model, professions are granted specific, protected occupational titles, and in some cases may be authorized to perform one or more health-related ‘controlled acts’ within their scope; controlled acts may be shared across professions [19].

In line with new legislation intended to govern each of the three professions, the Ontario government appointed a regulatory Transitional Council for each group, with the intention of laying the framework for each profession’s regulation in the public interest. In all cases, members of Transitional Councils were ‘chosen from individuals who responded to a public call for members advertised across Ontario’; Council members consisted of both practitioners from within the profession being regulated, as well as ‘general’ members who were not practitioners [20–22]. In the case of naturopaths, who had been previously regulated under another piece of provincial legislation, all Directors of the previous regulatory Board were included in the profession’s new Transitional Council [20].

We have previously characterized each of the three occupational groups as being at notably different stages of professionalization [10], which has contributed to different trajectories for each group’s regulatory process. Ontario’s naturopaths were the most tightly organized of the three, had achieved standardization in their training requirements many years earlier, and as noted above had been previously regulated in the province. Chinese medicine practitioners (whose practices include traditional acupuncture) had long lobbied for regulation, but were considerably divided as a community over the issue of professional standards. Homeopaths were similarly heterogeneous as a group, but were internally divided in terms of regulation as a goal, and had lobbied comparatively little for regulation.

Despite the significant differences in each group’s professional evolution, the Ontario government accepted the recommendations of the Health Professions Regulatory Advisory Council (http://www.hprac.org/en/index.asp) to take the step of regulating each of these groups around the same time. Regardless, the work of each group’s regulatory Transitional Council has been distinct, informed by the group’s specific needs moving forward. To date, Ontario’s Chinese medicine practitioners are the only group of the three that has fully implemented its new regulations (in April of 2013); naturopaths and homeopaths are expected to complete this process some time in 2015.

### The current study

Although the literature has extensively detailed TCAM practitioner groups’ interest in seeking professional legitimation, including some retrospective studies of such groups’ regulatory entry, we are unaware of studies detailing the transition of these groups through an *active* regulatory process. In an effort to capture the experiences and perspectives of Ontario naturopaths, homeopaths and Chinese medicine practitioners (including acupuncturists) as they passed through the transition to being regulated under the Regulated Health Professions Act, we initiated a cross-sectional survey in 2011, while all three groups’ regulatory Transitional Councils were part-way through their work. Our census-style surveys were mixed in methodological design: alongside numerous quantitative questions, they included one qualitative question inviting respondents to further expand their views on the emerging regulatory changes.

As will be elaborated elsewhere, and perhaps unsurprisingly given the three groups’ previously stated motives for pursuing regulation, the quantitative aspects of the survey demonstrated a widespread overall support for the proposed new regulations across all three practitioner groups; we intend to detail these results elsewhere. Across the three groups, many respondents also offered lengthy and, at times, impassioned written responses to our qualitative survey question, in some cases even attaching letters and newspaper clippings to their surveys in an effort to communicate their perspectives. What was notable across the responses was that, despite overwhelming support for the regulatory changes, members of all three groups felt ‘worried’ in varying degrees about the proposed regulations. In particular, such concerns pertained to issues of professional scope, fair registration standards, and a potential compromise to their groups’ underlying paradigmatic principles.

While some concerns were shared across the three TCAM practitioners groups, others were occupationally-distinct. This suggests that each group may have been facing particular but predictable challenges (e.g. consensus building around common standards) related to its current stage of professionalization. However, a number of the worries raised across the three groups appeared specifically related to the groups’ distinct identities as TCAM occupations; for example, some concerns appeared rooted in the groups’ relative marginality within the broader system of biomedical health professions. In addition, some concerns raised may be related to the implementation of Ontario’s overlapping practice scopes regulatory model, as will be discussed further on.

Regardless, the practitioner ‘worries’ raised in our study hold perhaps greatest significance in relation to the palpable challenge of regulating TCAM practitioners and practices hitherto largely excluded from national health care systems worldwide. As our study respondents clearly point out, it is one thing for a government to ‘decide’ to regulate, and quite another to unfold the details of such regulations in a way that is satisfying to various stakeholders. Any professional regulatory project will, for instance, invariably exclude some practitioners from entry; how to do so fairly and consistently may pose particular challenges in regulating TCAM practitioner groups. Cross-jurisdictional differences in regulatory scope may pose an additional challenge for some groups. The logistics of ‘grandparenting’ long-standing practitioners, navigation of cultural alongside clinical issues present in regulating traditional medicine practitioners, and the potential for producing new inter-professional hierarchies are additional issues which study respondents raised in Ontario’s context. Many such issues will likely arise in other jurisdictions as well. As such, this work may inform the global conversation on TCAM regulatory initiatives, while raising important questions for further scholarly investigation.

## Methods

In 2011 and 2012, we distributed an online survey to naturopaths (*n* = 882), homeopaths (*n* = 784), and both online and print surveys to Chinese medicine practitioners or acupuncturists (*n* = 1286) across Ontario using a census model whereby we aimed to reach as many practitioners as possible. Prior to sending out the surveys, we had undertaken an extensive process to gather practitioner names and contact information. The naturopath practitioner population was contracted through the membership lists from the Ontario Association of Naturopathic Doctors (OAND), the largest professional association for NDs in Ontario. The OAND sent an email with a link to the survey to their members (*n* = 882). The Transitional Council of the College of Homeopaths of Ontario (TC-CHO) provided a list of homeopathic practitioners interested in receiving information about regulation and we supplemented this with further contacts generated through personal connections and internet searches (*n* = 784). Finally, Chinese medicine practitioners were identified from practitioner organizations’ publicly-accessible member lists, internet searches using Google and online indexes, such as the Yellow Pages, Chinese newspapers, and personal visits to Chinese business centres in the greater Toronto area. Based on pre-test information, we determined that the survey should be offered in both English and traditional Chinese language, as well as accessible in both online and paper versions. The questionnaire, introductory letters, and reminders were translated into traditional Chinese by a professional translator and then back translated to check for accuracy. Respondents were then contacted by email to fill out the online survey (*n* = 659) and by post mail for the paper survey (*n* = 627). Initial response rates across the three groups were 50 % (naturopaths), 56 % (homeopaths), and 37.5 % (Chinese medicine practitioners).

Because both Chinese medicine practitioners and homeopaths were not regulated at the time of our study, any individual self-identifying as a practicing Chinese medicine practitioner or homeopath was included in the original sampling frame, regardless of their current education or work status. However, once our sample was compiled, the data were limited to include only those practicing in Ontario on a full or part-time basis (this also excluded some naturopaths). Furthermore, for Chinese medicine practitioners, respondents already registered in Ontario under another regulated health profession (physiotherapists, nurses, MDs, etc.) where acupuncture is included as part of their scope of practice were excluded from our study (*n* = 111), given our focus on those who must register as a Chinese medicine practitioner to practice acupuncture. These exclusions left us with a final sample of 1047, broken down as 427 naturopaths, 329 homeopaths and 291 Chinese medicine pracititoners. Of these respondents, a sub-sample of 273 naturopaths (64 %), 234 homeopaths (71 %) and 181 Chinese medicine practitioners (63 %) provided responses to the qualitative question in our study, which is the focus of this paper. The study received ethical approval from the Research Ethics Board at the University of Toronto.

The key demographics describing the groups are presented in Table 1.^1^ From these results, we can see that women are most predominant among naturopaths (79 %) and least so among Chinese medicine practitioners (57 %). Additionally, naturopaths report an average age of approximately 38 years, while homeopaths and Chinese medicine practitioners have mean ages of 47 and 49 years, respectively. With respect to years in practice, Chinese medicine practitioners have been practicing the longest at approximately 17 years, while naturopaths report an average of 8 years of experience and homeopaths nine. Finally, a little over half of naturopaths report working part-time compared to 65 % of homeopaths and 34 % of Chinese medicine practitioners.^2^

**Table 1:** Demographic characteristics by group for qualitative respondents

	Naturopaths (*n* = 273)	S.D.	Response Rate	Homeopaths (*n* = 234)	S.D.	Response Rate	CMP^c^ (*n* = 181)	S.D.	Response Rate
Gender^a^	0.793	-	99 %	0.740	-	99 %	0.572	-	99 %
Age	38.3	9.79	97 %	47.1	10.6	99 %	48.7	11.6	96 %
Years in practice	8.06	7.49	100 %	9.29	8.89	99 %	17.3	12.01	99 %
Part-time^b^	0.520	0.501	100 %	0.645	0.479	100 %	0.337	0.474	100 %

^a^0 = Men, 1 = Women

^b^Reference category is full-time

^c^Chinese medicine practitioners

The surveys contained a range of questions focused on the demographic, practice and educational characteristics of practitioners, with some questions common across surveys and some reflecting particular aspects of each group. After a series of closed-ended questions related to regulation, respondents were asked a broad question to solicit their views on the regulatory changes facing their occupational group. Homeopaths, for instance, were asked: *Finally, what are your thoughts about the decision to regulate homeopaths under the Regulated Health Practitioners Act in Ontario?* Questions posed to the other groups were very similar, with the practitioner group name appropriate replaced. Qualitative responses from across the three surveys were inductively coded by two independent coders for emergent themes. The two coders compared and corroborated coded findings for consistency and reliability. Themes emerging across the three groups were then compared to find areas of overlap and uniqueness.

## Results

Overall, written responses from across the three surveyed groups affirmed quantitative findings showing significant support for regulation from about three-quarters of all respondents across the three groups. A more extensive analysis of quantitative findings will be reported elsewhere. Written survey responses suggested that this support reflected respondents’ perceptions that the regulatory changes would enhance their occupations’ credibility, increase availability of third party insurance coverage for their services, and help protect the public from untrained practitioners. However, despite this widespread support for the regulatory changes in principle, quantitative findings across the naturopathic, homeopathic and Chinese medicine practitioner groups also showed that a considerable proportion of respondents (48, 44 and 33 % respectively) agreed or strongly agreed when asked to respond to the following statement: “I am worried about regulation”.

The substance of such ‘worries’ is difficult to evaluate from the quantitative findings alone, results of which are limited by the specific questions asked. However, thematic analysis of written survey responses from naturopathic [N], homeopathic [H] and Chinese medicine [CM] practitioners proved useful in drawing out a number of key regulation-related concerns, some of which were common across the three groups, and others being occupation-specific.

Some respondents who expressed concerns about the regulatory changes were in fact opposed to these changes, characterizing it, for instance, as “not a good idea” [CM]. A small number of practitioners argued that a discourse emphasizing public safety was being disingenuously bandied about as a political pretext for increased regulatory control over low-risk clinical activities, for instance:

- *One regulates activities that are dangerous. Homeopathy is not dangerous.* [H]

Others felt poorly informed about the regulatory changes, and had difficulty understanding how it would affect them:

- *I feel that I am uninformed about the change in regulation and find the information that is provided to be confusing.* [N]

However, the majority of those expressing ‘worries’ did in fact support the new regulations, but had concerns or felt unsure about the way in which these were being implemented:

- *I do support it but don’t know what will be the real outcome. Hopefully it will be a positive one to both public and practitioners.* [CM]
- *I feel this regulation has been long overdue. Now that the opportunity has come about I am disappointed in the quality of the regulations proposed.* [N]

More specifically, respondents from all three groups expressed concerns with the actions of the Transitional Councils responsible for drafting their respective new regulations:

- *After reviewing the Draft regulation put out by the Transitional council, my support for regulation diminished almost immediately.* [H]
- *I am not confident that all of those who are representing my profession are acting appropriately on my behalf.* [N]

Within the Chinese medicine group, respondents raised concerns about the composition of their particular Transitional Council:

- *My only wish is to have authentic TCM and acupuncture professionals involved in the regulations to ensure fairness, equitableness [sic], and openness.* [CM]

Overall, respondents’ ‘worries’ cut across four primary themes, most of which were broadly shared across the three groups, though with occupationally-specific components. These four themes involved concerns that the new regulations might: a) produce an unwanted financial and administrative burden on practitioners; b) detrimentally affect groups’ practice scopes; and c) implement inappropriate or unfair registration standards; and d) compromise occupational groups’ paradigmatic foundations.

## Increased administrative and financial burden

A number of respondents who otherwise supported the regulatory changes raised concerns that these changes might increase administrative work for practitioners, and potentially cause negative financial impacts:

- *Worried about added costs, bureaucracy and paper work headaches but am generally supportive of this measure to further professionalize our profession.* [CM]

Several respondents raised financial concerns within the broader context of monetary struggles within their occupational group:

- *It will…likely raise my registration dues, which will make practicing unaffordable to myself who is a newer practitioner. *[N]

Others concurrently connected such concerns with the issue of providing financially accessible services to patients:

- *Regulation will cause more expenses for the Homeopath, thus making it more and more difficult for a Homeopath to make a viable living. Regulation will cause rate increases thus reducing affordability to the average family/public.* [H]

Some respondents even predicted that “regulation costs” (amongst other factors) could “cause a lot of practicing homeopaths to go underground/practice without licensing” [H].

## Negative impact on practice scopes

Respondents from all three groups worried that the new regulations might have detrimental impacts related to their specific practice scopes. Where concerns about *reduced* scope predominated naturopaths’ comments, it was a concern around *overlapping* scopes that repeatedly appeared in homeopaths’ and Chinese medicine practitioners’ responses. Common across all three groups’ scope-related concerns were allusions to both inter- and intra-occupational turf battles.

As noted above, naturopathic respondents repeatedly expressed concern that their existing practice scope would be reduced under the new regulations:

- *If the intent of the regulations is to evolve into a better regulated profession it cannot be at the cost of decreasing our scope of practice.* [N]

Furthermore, several naturopaths expressed a concern that the new regulations might not expand their scope adequately. Specifically, such respondents hoped that naturopaths’ diagnostic rights would be broadened to include particular biomedical tests (such as “Xray, MRI, CT scans” [N]), and the ability to deliver “intravenous therapies” and “prescribe base-line pharmaceuticals” as well as specialized supplements such as “hesperidin” [N] and “L-carnitine” [N].

Some naturopaths connected their scope-related concerns to a goal of practising as independent, primary care practitioners, on par with biomedical physicians:

- *My inability to directly order lab tests [sic] or prescribe base-line pharmaceuticals inhibits me from being a one stop shop for primary care. I have to rely on walk-in clinic MDs to offer complete care to my patients.* [N]

Others felt that the practice scope proposed in Ontario’s emerging naturopathic regulatory structure would disadvantage the province’s naturopaths in relation to other North American jurisdictions:

- *I feel the change will make us the laughing stock of medical providers in Ontario and we are already being scoffed at by other ND’s across the country. It’s time for a national Naturopathic Regulation at the level of British Columbia and with the scope of Arizona.* [N]

However, not all respondents favoured all aspects of a regulatory scope of practice, particularly with respect to pharmaceutical drugs:

- *I have no desire to prescribe drugs and giving us this right invalidates the meaning behind our profession and principles.* [N]

Like naturopaths, some Chinese medicine practitioners - who otherwise supported the regulatory changes - also expressed concern that their scope would be limited under the new regulations:

- *[Regulation] is necessary. I am looking forward to my services being covered under supplementary health insurance. I am very concerned about restrictions in my practice.* [CM]

More specifically, such concerns related to particular components of Chinese massage practice (Tuina), which involve techniques similar to spinal manipulations used by chiropractors, which are restricted under Ontario’s ‘controlled acts’ model.

Amongst homeopaths and Chinese medicine practitioners, qualitative scope-related responses largely reflected a concern that the regulations would continue to permit other occupational groups to perform their respective core practices. Homeopaths specifically raised concerns about naturopaths’ continued authority to practice homeopathy:

- *I do not understand how homeopaths with extensive training in homeopathic remedies are not able to prescribe “restricted” remedies, yet naturopaths that only study 28 h[ours]of homeopathics have license to prescribe these restricted remedies when they have little understanding of how these remedies work.* [H]

Several homeopaths furthermore deplored that unlike naturopaths, they were not expecting to be granted a ‘doctor’ title in their professional legislation, presumably further undermining their relative credibility:

- *I am upset that we will be deemed as secondary health care practitioners underneath naturopaths, with our designation of Doctor of Homeopathy being withdrawn to Homeopath.* [H]

Similarly, numerous Chinese medicine practitioners raised concerns that non-Chinese medicine based health care professionals (such as physiotherapists, chiropractors and naturopaths) would retain the right to practise acupuncture within their professional scope. Many expressed a perception that such professionals were less qualified to practise acupuncture so than traditional acupuncture practitioners:

- *I am in favor of regulation for the profession, I wish that it was also more restricted to fully qualified/future fully registered with the college practitioners instead of being allowed to be practiced in other fields as Chirop[ractic] … who do not receive or are required to have proper education and yet will be able to practice acupuncture within their scope of practice and profession.* [CM]

Evidently such concerns around overlapping practice scopes relate to the issue of training standards, which will be discussed in greater detail further on. However, the question of regulatory turf repeatedly emerged as well, with several homeopaths and Chinese medicine practitioners expressing a wish - as described above - to see their core practice scopes more exclusively restricted.

Some Chinese medicine practitioners accepted that they would continue, within Ontario’s regulatory context, to share some core practices such as acupuncture with other professions. However, they wished to see the term ‘acupuncture’ protected for Chinese medicine practitioners under the regulations, thus differentiating traditional acupuncture from more biomedically-informed needling techniques, which one Chinese medicine respondent proposed be termed “intramuscular stimulation or dry needling” [CM] instead of ‘acupuncture’.

## Inappropriate or unfair registration standards

Amongst homeopaths and Chinese medicine practitioners, groups for whom there were (at the time) no national educational or regulatory standards, considerable concerns were raised as to how the new regulations might assess practitioners’ qualifications for professional entry. Respondents across the two groups tended to agree in principle that some practitioners’ current training within their occupational groups were “not high enough” [H]; and that the enforcement of standards would likely “improve the quality of care to patients” by increasing “the practitioners’ level of treatment” [CM]. However, a concern around how such standards might be set - particularly in light of significant intra-occupational diversity - permeated many respondents’ comments:

- *I am hoping that there will be a fair way of assessing each of our training as there are many ways to practice that benefit the patient.* [H]

In particular, respondents across both homeopathic and Chinese medicine groups raised concern that various practitioner subgroups might be *inappropriately* excluded from registration, depending on how the standards were set. However, each group’s individual concerns in this regard were more occupationally-specific.

### Homeopaths

Amongst homeopathic respondents, standards-related comments were dominated by the question of how much biomedical scientific training should be required of practitioners in the forthcoming registration standards. Those advocating for more extensive biomedical training argued it would produce higher competency within, and greater credibility for, the homeopathic community:

- *[U]ntil we come together as a scientific community where those who wish to practice homeopathy are required to attain a significant and competent knowledge of the hard sciences and even the soft sciences (biology, nutrition, physiology, etc..), credibility will suffer whether we regulate or not.* [H]

Others de-emphasized the importance of biomedical scientific training in the professional development of competent homeopaths:

- *I think regulation is good in that, only those homeopaths who have been properly trained will be permitted to practice. I don’t think, however, that rigorous training in the health sciences is necessary to be a good homeopath.* [H]

Overall, such comments illustrated a clear lack of consensus on this issue, and worry within disparate homeopathic ‘camps’ as to how the standards would eventually emerge to determine what - or who - would be included. Several respondents feared that those representing a more “classical, Hahnemannian homeopathy” [H] would not get their voices heard in this regard, perceiving that the regulatory process had thus far been dominated by “a minority of homeopaths” [H].

A number of homeopaths also raised concern that the standards proposed for ‘grandparenting' long-standing practitioners under the new regulations might inappropriately exclude those practising part-time and in rural areas, new graduates, and practitioners with overseas training:

- *[T]he number of hours proposed as a minimum to qualify for registration are unrealistically high and not reflective of the difficulties of introducing this unknown form of medicine to the public - particularly in semi-rural and rural areas.* [H]
- *…the drafts as prepared to date… will likely preclude the possibility of the majority of recent homeopathic graduates from becoming registered.* [H]
- *I am concerned if I will be accepted by the Act because I studied in South Africa.* [H]

### Chinese medicine practitioners

Some Chinese medicine practitioners, by contrast, worried that the standards had been too-narrowly set, excluding those practising East Asian varieties of traditional acupuncture falling outside of the Chinese tradition:

- *My understanding is that [the regulation] focuses only on the Chinese tradition which is a mistake. It should include all Qi-based medicine and specialties like Korean and Japanese acupuncture.* [CM]

Others worried that those who had been trained via apprenticeship or family lineage, which one respondent termed “the special path of studying TCM and acupuncture” [CM] might not be eligible for registration. Also raised were the potential challenges long-standing practitioners might face in collecting documentation of their training for the grandparented registration process:

- *Asking for transcripts or study reports may [create] great difficulties for those old practitioners, for these schools, or professors may not exist anymore [sic].* [CM]

A small number of respondents opposed the grandparented registration process outright, arguing that all registrants should be required to

- *[W]rite an exam in order to have a legal license!…no Grandfather law.* [CM]

However, Chinese medicine practitioners’ most pronounced concern around grandparented registration pertained to the issue of language. Numerous practitioners asserted that the regulations should permit practitioners not only to permitted to conduct their clinical practices in a Chinese language, but also to complete registration examinations and keep patient records exclusively in Chinese. Several respondents raised significant alarm that they might be excluded from registration if the regulations enforced English (or French) as the profession’s primary language. For some individuals, such concerns had entirely dissuaded them for supporting the regulations as currently proposed:

- *Current legislation is biased towards eliminating TCM doctors, ousting TCM doctors. I oppose. Was taught TCM by grandfather. I don’t know English. Grand-fathered regulation requires English testing. I am on the verge of being eliminated.* [CM]

The potential compromise that the new regulations posed in terms of occupationally-specific values - whether cultural or otherwise - was an issue raised by practitioners across the three surveyed groups.

## Compromise to occupational paradigm

A subgroup of respondents from across all three groups expressed concern that the regulatory changes threatened to compromise their respective occupations’ underlying paradigmatic foundations, despite the changes being perceived as positive in other respects. Within the Chinese medicine and homeopathy groups, practitioners expressed concern that their paradigmatically-distinct practices were being forced into a regulatory structure designed to accommodate professions more fundamentally rooted in biomedicine:

- *We should not try to ‘fit homeopathy’ into a set of regulations designed for other professions.* [H]
- *Regulation of TCM/acupuncture should be based on TCM/acupuncture’s unique Chinese medical nuances. It should not blindly follow the regulatory methods of Western medicine.* [CM]

Several homeopathic respondents expressed concern that under the new regulations, their profession would become “more medical and less homeopathic” [H], thereby compromising the “spirit of homeopathy” [H]. Similarly, a number of naturopaths characterized the regulatory changes as part of a trend “moving away from our true purpose as nature doctors” [N], and “treating more like Green MDs” [N]. They described a shift within naturopathy away from a “drugless therapy” [N] scope of practice to “more of an allopathic medical scope of practice” [N], which emphasized “treating the disease, instead of the individual” [N]. According to some practitioners, such shifts in naturopathy’s focus were arising from a regrettable “need for approval from the conventional medical world” [N].

Within the Chinese medicine group, several respondents expressed concern that regulators were not adequately taking into account factors related to “culture and tradition” [CM], ultimately threatening to erode the occupation’s paradigmatic foundations, and with it the quality of Chinese medicine care. These detrimental effects would, they argued, detrimentally impact patients, especially people of East Asian origin using Chinese medicine as their predominant form of health care. For example:

- *[Decision makers] don’t know this professional field so their decisions cannot reflect real TCM [traditional Chinese medicine] practitioners’ thought and practice. If this regulation is passed it will definitely kill TCM’s long-term practice in Ontario. The public who heavily depend on Chinese medicine will suffer.* [CM]

It is clear that respondents from across all three occupational groups - naturopaths, homeopaths and Chinese medicine practitioners - expressed multiple ‘worries’ about the way in which Ontario’s new regulations governing their work would be implemented. Some concerns reflected fundamental issues relating to each group’s underlying worldview and core practices; others pertained to more immediate financial or administrative concerns. It is important to note that for the majority of practitioners surveyed, such ‘worries’ appeared notably secondary to an overarching spirit of support for Ontario’s new regulations governing each of them. Nevertheless, the expressed ‘worries’ do warrant careful consideration in relation to the broader global issues surrounding regulation of traditional, complementary and alternative medicine (TCAM) practitioner groups.

## Discussion

In several ways, practitioners’ accounts of moving towards regulatory inclusion in this study appear to describe a less idealized experience than many might have hoped, echoing previous studies pointing to the tradeoffs inherent in TCAM groups’ professionalization projects [2–4]. Some respondents’ concerns that regulation might compromise their occupations’ paradigmatic foundations are not new: the threats of occupational co-optation within a biomedically-dominant health care system (in which institutionalized training is highly valued) have been extensively discussed [2, 3, 23, 24]. Whereas some recent studies suggest that institutionalization of some TCAM practitioner trainings may permit considerable inclusion of holistic philosophical content at odds with biomedical epistemologies [25, 26], other work has noted that institutionalization tends to promote increased biomedicalization of TCAM trainings [27, 28]. Regardless, another potential challenge for TCAM practitioners to face is that any regulatory process designed to implement stringent registration standards will necessarily exclude some applicants. However, the varied ‘worries’ raised in our study appear in several ways to move beyond such previously reported issues.

Of particular relevance in our findings are practitioner fears about unjust exclusion of specific practitioner subgroups, whether part-time or foreign-trained practitioners, immigrants, those lacking English fluency, or those trained through traditional apprenticeship. The professions literature does indeed document inequities inherent in many regulatory bodies’ registration practices [29, 30], and it remains to be seen how effectively Ontario’s TCAM professional regulatory bodies are able to first define, and subsequently implement, fair registration practices. As Ontario’s provincial Fairness Commissioner - a government body responsible for ensuring equitable registration requirements across the health professions - has noted, regulation of professions like homeopathy and Chinese medicine, ‘that have their roots in traditional ways of knowing,’ raises unique challenges from a regulatory equity perspective [31]. In particular, the issue of regulatory grandparenting strategies - a commonly engaged short-term strategy for bringing long-standing practitioners under the auspices of a new professional regulatory structure [32] - may warrant significant additional research in relation to TCAM practitioners groups. Indeed, while some studies have described the details of grandparenting strategies for TCAM practitioner groups [32–34], little sociological analysis has been applied to such approaches.

In addition to such equity-related points, our findings highlight the significant internal diversity characterizing particular TCAM occupational groups, in which distinct subgroups may hold notably different views about regulation and its implementation, as well – in particular – as appropriate educational standards. Although such internal diversity has been previously pointed out [3, 10, 15], its dimensions and implications within and across various TCAM practitioner groups may warrant further scholarly exploration.

Moving to the distinct ways in which each occupational group framed its particular ‘worries’ about regulation, it is possible that such distinctions may have been influenced by each group’s stage of professionalization at the time when they were granted regulatory status. As we noted in our earlier study of the same occupations, naturopaths represented the most advanced stage of professionalization of the three groups, having previously established clear, cross-jurisdictional standards and a distinct professional identity. That naturopaths surveyed in the current study articulated few standards-related concerns but focused instead on achieving a broad regulatory practice scope on par with other North American jurisdictions is thus not surprising.

Chinese medicine practitioners, whom we characterized in our 2004 study as internally fragmented over the degree of biomedical sciences that should be included in occupational standards, appeared to have since moved beyond this concern as a community, instead focusing on a more nuanced set of tensions between acupuncture as a clinical practice, and the importance of Chinese medicine’s cultural/historical dimensions. This focus is reflected in practitioner concerns around language proficiency requirements, recognition of practitioners trained by apprenticeship, and the question of ‘which’ East Asian acupuncture styles should be explicitly validated in Ontario's regulations. These issues – which our research team is currently investigating further – raise a number of complex regulatory questions which may be unique to traditional medicine occupational groups, for whom both clinical and cultural concerns may hold parallel importance. As several survey respondents suggest, the question of regulatory English proficiency requirements may not, for instance, be adequately addressed as a public safety issue alone, but appears to raise a range of complex historical and cultural dimensions related to preserving the traditional East Asian medicine system’s roots within a western liberal democratic regulatory structure.

Homeopaths, who in our previous study were the smallest and least organized of the three group, and arguably the least ‘ready’ for regulation, continued to struggle internally over the issue of biomedical scientific training even as Ontario granted them self-regulatory status: a concern which had similarly dominated our earlier discussions with community leaders. It is perhaps self-evident that biomedicine is currently the ‘lingua franca’ in widespread usage between regulated health professions, and that TCAM professions’ adoption of biomedical language and perspectives may indeed facilitate their participation in mainstream health care systems. However, as Hollenberg and Muzzin have pointed out [24], the increased biomedicalization of TCAM professions is neither socially nor politically neutral, but may better be understood within the related historical contexts of increased biomedical dominance in health care worldwide. As such, the process of TCAM professions ‘integrating’ into mainstream health care necessarily raises complex questions of power; and TCAM groups’ concerns that they may be losing their core identities in a process of ‘enforced assimilation’ should be further investigated in this light. Furthermore, it is worth noting that some TCAM occupations (e.g. naturopathy, chiropractic) appear to have more enthusiastically adopted biomedical theory and terminology as essential to implementation of their therapeutic systems than have others (e.g. Chinese medicine, homeopathy, Ayurveda), which may have considerable sociological and political consequences.

Homeopaths’ and Chinese medicine practitioners’ concerns around having to ‘share’ some of their key clinical practices with other professions had not appeared in our earlier studies. These concerns may have simply reflected some practitioners’ poor understanding of Ontario’s health professions regulatory structure, which governs ‘controlled acts’ but does not authorize exclusive practice scopes. This in itself is an important issue, emphasizing a potential challenge regulators may face in effectively communicating with TCAM practitioners through a regulatory process. It is unclear whether such communication challenges are similar to those otherwise faced by government bodies in other pursuits, or if there are more TCAM-specific issues at play in this regard. However, more broadly contextualized, the concerns raised by members of both of these occupational groups about overlapping practice scopes may also speak to larger trends at play in the field of TCAM professional regulation.

For example, some Ontario Chinese medicine practitioners’ concern that under the new regulations they would continue to ‘share acupuncture’ with other professions significantly echoes ongoing professional turf battles over acupuncture across the non-East Asian world [35]. In the United States, for example, two recent court cases launched by traditional East Asian medicine practitioners in the states of Oregon [36] and Washington [37], have ruled that chiropractors and physiotherapists, respectively, may no longer engage in acupuncture-like ‘needling’ practices within their scopes. Although these states’ regulatory structures are certainly different from Ontario’s in that they employ licensure models with exclusive practice scopes, it remains noteworthy that such inter-occupational turf battles over acupuncture are increasingly common. Elsewhere, in Australia, where Chinese medicine practitioners have recently been granted the protected title of ‘acupuncturist’ and exclusive professional authority to describe their work as ‘acupuncture’, the act of inserting thin needles for therapeutic purposes remains in the public domain, that is, not exclusive to this occupational group. As such, members of several other professions have ‘rebranded’ their needling activities using such nomenclature as ‘dry needling’ and ‘intramuscular stimulation’ [38], raising the ire of East Asian acupuncturists. Although an Australian regulatory impact analysis had previously determined that limiting acupuncture practice to East Asian medicine practitioners was unlikely to produce public benefit [39], an early Ontario government analysis of similar issues (which was later abandoned) had reached considerably different conclusions [40]. Aside from the vital question of public safety in this regard, there are several additional dimensions warranting consideration in this regard. For instance, various occupations will not only occupy distinct positions within an interprofessional hierarchy/ecology [41, 42], but will also approach a particular practice (such as acupuncture) from distinct epistemological foundations.

Of similar interest is the concern raised by several homeopaths that they would be sharing the practice scope of homeopathy with Ontario’s naturopaths. To be clear, the practice of recommending homeopathic remedies has not been designated as a ‘controlled act’ under Ontario’s Regulated Health Professions Act. As such, the practice remains in public domain; and no limitations exist in Canada on the sale of homeopathic remedies themselves, as long as they meet the country’s federal requirements governing natural health products. Regardless, what is poignant in some homeopaths’ accounts is a perception that the new regulations may secure for the homeopathic group a subordinate sociopolitical position in relation to the naturopathic profession. Indeed, the regulations will at once permit both naturopaths and homeopaths to ‘practice homeopathy’ within their professional scopes, while requiring less homeopathy-specific training for naturopaths, and granting the coveted ‘doctor title’ to naturopaths and not homeopaths, the apparent ‘homeopathy experts’. Two points are significant in this regard.

First, in terms of regulatory structures, these claims raise questions about what constitutes competency in relation to Ontario’s ‘overlapping-scopes’ regulatory structure. This question, which arose both in relation to ‘homeopathic scope’ and with regards ‘acupuncture scope’ across various professions in our study, is important from a policy perspective. Indeed, an overlapping-scopes regulatory model has been increasingly adopted across Canadian jurisdictions, but little research has explored the nuances of its implementation or impacts since O’Reilly’s pioneering study describing the model’s emergence in Ontario [19]. Furthermore, interest in adopting overlapping-scopes regulatory models has been growing in the United States since the publication of the 1995 Pew Report on Reforming Health Care Regulation [43]. The Pew Report recommended phasing out regulatory structures based on exclusive practice scopes which promote professional monopolies that ‘unnecessarily restrict other professions from providing competent, effective and accessible care’ [44].

If overlapping-scopes regulatory models are to become more normative in North America, it may become important for regulators to engage more directly with clear criteria for what constitutes competent or effective care for specific practices. Such engagement may present several challenges. Within a self-regulatory context such as Ontario’s, it is typically professions themselves – rather than the state – which establish training standards. Moreover, even outside of a self-regulatory context, any state engagement with standards would likely require considerable input from those with expertise in the relevant fields – namely members of the scope-overlapping professions themselves. The challenge of balancing each profession’s self-interest with the public interest through this process may require considerable negotiation. This may be particularly challenging in regulating TCAM professions and practices within biomedically-dominant regulatory frameworks, as high quality scientific evidence for TCAM therapies is in some cases just emerging; and because some practitioner groups may draw in varying degrees on non-biomedical evidentiary paradigms. Governments may consider adopting the World Health Organization’s educational standards guidelines for various TCAM practices to assist in this type of groundbreaking regulatory work.

The second point of interest around the homeopathy/naturopathy tension suggested in some respondents’ comments in our study is the issue of inter-professional stratification under a regulatory model that was originally intended to ‘level the playing field’ between professions, rather than fortify additional hierarchies between them [19]. The sociological professions literature has detailed the subordination of TCAM practitioner groups in relation to biomedical physicians [3, 7, 12], but to date little work has addressed the unequal power relations that may be emerging between TCAM groups as they move to professionalize.

## Conclusions

In conclusion, the current study raises several key theoretical and practical points while providing a unique snapshot of practitioner perspectives across three distinct TCAM professions as they pass through a long-awaited transition into state-regulated status. The advantage of our study’s census survey model is that it offered a broad cross-section of respondents with an opportunity to contribute their perspectives. Having undertaken an extensive process to gather practitioner names and contact information across multiple media, our search was quite comprehensive. Nevertheless, some practitioners may have been inadvertently missed. That said, the broad range of perspectives exposed through inductive analysis of participants’ responses to an open-ended question in a predominantly quantitative survey (quantitative findings discussed elsewhere) represent an important series of insights, which may not have otherwise been exposed [45], thus highlighting the potential scholarly value of a rigorous, mixed-methods approach.

Regardless, this work does have limitations. Some respondents’ opinions, for instance, may have been based on incorrect information either about Ontario’s regulatory structure, about the details of their occupation’s regulatory process, or both. We furthermore recognize the limitations of collecting robust qualitative data using a survey format as compared, for example, to conducting in-depth interviews, which invariably provide more detailed information with greater nuance. Regardless, that so many survey respondents gave lengthy and detailed written comments in response to a single qualitative question posed in a predominantly quantitative survey, suggested a high level of practitioner engagement in the regulatory project, which we considered significant.

It remains to be seen to what degree the diverse ‘worries’ described by Ontario homeopaths, naturopaths and Chinese medicine practitioners experiencing an important milestone in their professional projects shall endure as the regulations take effect; or whether such concerns more simply reflect the inevitable transitional discomforts that may be expected to accompany the impacts of a significant regulatory change. We will continue to follow these issues as these groups evolve into established regulated professionals in the province of Ontario. In the interim, it is our hope that the perspectives explored in this work may help to illuminate the internal struggles faced globally by TCAM practitioner groups passing through regulatory transitions, and to inform further investigation around how such regulations may be more effectively and seamlessly implemented, in the public interest, across nations.

## Footnotes

1 Not all qualitative respondents provided demographic data for all key variables, so within group response rates are listed for each demographic variable in Table 1.

2 Post-hoc tests were conducted within each group to determine whether those who responded to the qualitative question differed significantly on these key demographic characteristics as compared to those who did not answer the qualitative question. For naturopaths, these results showed that qualitative respondents are older and less likely to be women than non-respondents. For both homeopaths and Chinese medicine practitioners, the only difference is with respect to age: qualitative respondents are older than non-qualitative respondents. There are no differences in any group with respect to years in practice or part-time versus full-time status.
